# Tissue Restricted Splice Junctions Originate Not Only from Tissue-Specific Gene Loci, but Gene Loci with a Broad Pattern of Expression

**DOI:** 10.1371/journal.pone.0144302

**Published:** 2015-12-29

**Authors:** Matthew S. Hestand, Zheng Zeng, Stephen J. Coleman, Jinze Liu, James N. MacLeod

**Affiliations:** 1 Gluck Equine Research Center, Department of Veterinary Science, University of Kentucky, Lexington, KY, United States of America; 2 Department of Computer Science, University of Kentucky, Lexington, KY, United States of America; Florida Atlantic University, UNITED STATES

## Abstract

Cellular mechanisms that achieve protein diversity in eukaryotes are multifaceted, including transcriptional components such as RNA splicing. Through alternative splicing, a single protein-coding gene can generate multiple mRNA transcripts and protein isoforms, some of which are tissue-specific. We have conducted qualitative and quantitative analyses of the Bodymap 2.0 messenger RNA-sequencing data from 16 human tissue samples and identified 209,363 splice junctions. Of these, 22,231 (10.6%) were not previously annotated and 21,650 (10.3%) were expressed in a tissue-restricted pattern. Tissue-restricted alternative splicing was found to be widespread, with approximately 65% of expressed multi-exon genes containing at least one tissue-specific splice junction. Interestingly, we observed many tissue-specific splice junctions not only in genes expressed in one or a few tissues, but also from gene loci with a broad pattern of expression.

## Introduction

With several fold more proteins in the human proteome than the number of protein-coding genes in the human genome, most gene loci clearly serve as a template for multiple protein variants. A transcriptional process that is widely recognized as a major contributor to proteome complexity is alternative splicing, in which different exon splicing patterns generate multiple mRNA transcript structures from the same gene locus. The functional importance of exon splicing in cell biology has been demonstrated in a number of systems. For example, in Drosophila alternative splicing is an important regulatory mechanism involved in such diverse processes as sex-determination, muscle type specificity, and nervous system development, and can be found in a variety of genes: from ion channel encoding genes to transcription factors (reviewed in [1]).

Recent estimates suggest that as many as 95% of multi-exon genes are alternatively spliced in mammals, often displaying tissue-specific patterns [2, 3]. With 15–50% of human disease mutations affecting splicing [4], it is crucial from a medical standpoint to understand normal splicing patterns in healthy tissue. Indeed, altered splicing has been associated with myotonic dystrophy [5], spinal muscular atrophy [6], Hutchinson-Gilford progeria syndrome [7], familial dysautonomia [8], and many cancers (including common cancers, such as colon [9, 10] and breast [11] cancers) (reviewed in [12]).

Gene structure and splicing patterns have traditionally been determined through conventional Sanger sequencing and the alignment of long reads (mRNAs or ESTs) to a reference genome. This method is well suited for a focused assessment of individual genes, but is generally too cumbersome for investigating transcript structures from thousands of gene loci in parallel. Next-generation sequencing machines, such as the HiSeq by Illumina, generate millions of short reads in a fraction of the time and cost. Therefore, next-generation sequencing of mRNA fragments (RNA-seq [13]) makes gene annotation much more affordable in terms of money and time. However, difficulties have arisen in identifying exon structures and constructing full length transcripts from short-read data. Current estimates of sensitivity and precision for determining exon structures, and connecting them into transcripts, tends to be below 75% [14, 15]. Alternatively, third generation sequencers, such as the PacBio RSII by Pacific Biosciences, have long sequence reads that can fully span a transcript, simplifying transcript structure identification. However, these platforms have length biases and lack the sequencing capacity to properly detect very short or very long transcripts, as well as low expressed genes [16, 17]. Short read technologies have been shown to detect many of the splice-junctions identified in long read technologies, as well as have high similarity with previously annotated splice-junctions [17, 18]. Therefore, we focus this study on identifying splice-junctions expressed in specific tissues using high-throughput short-read data.

In this study, we compare RNA-seq data from 16 different human tissues to identify annotated and novel internal splice sites, their expression levels, and annotated gene expression levels. The analysis enabled a distribution assessment of splice junction and gene expression across different tissues, including a comparison between the levels of tissue specificity for restricted patterns of splice junctions, gene expression, and their association.

## Results

### Sequence alignment

Bodymap 2.0 RNA-seq data from 16 normal human tissues were used to identify tissue-specific splice junctions. The individual tissues provided an average of 79 million single end and 160 million paired-end reads (considering each end as a separate read). Seventy-five to eighty-four percent (80% on average) of all single end reads per tissue aligned uniquely and 4–8% (6% on average) aligned non-uniquely to the human genome. For the paired-end reads, 71–83% (78% on average) aligned uniquely on average per tissue and 1–3% (2% on average) aligned non-uniquely.

### Splice junction evaluation

**Fig 1:**
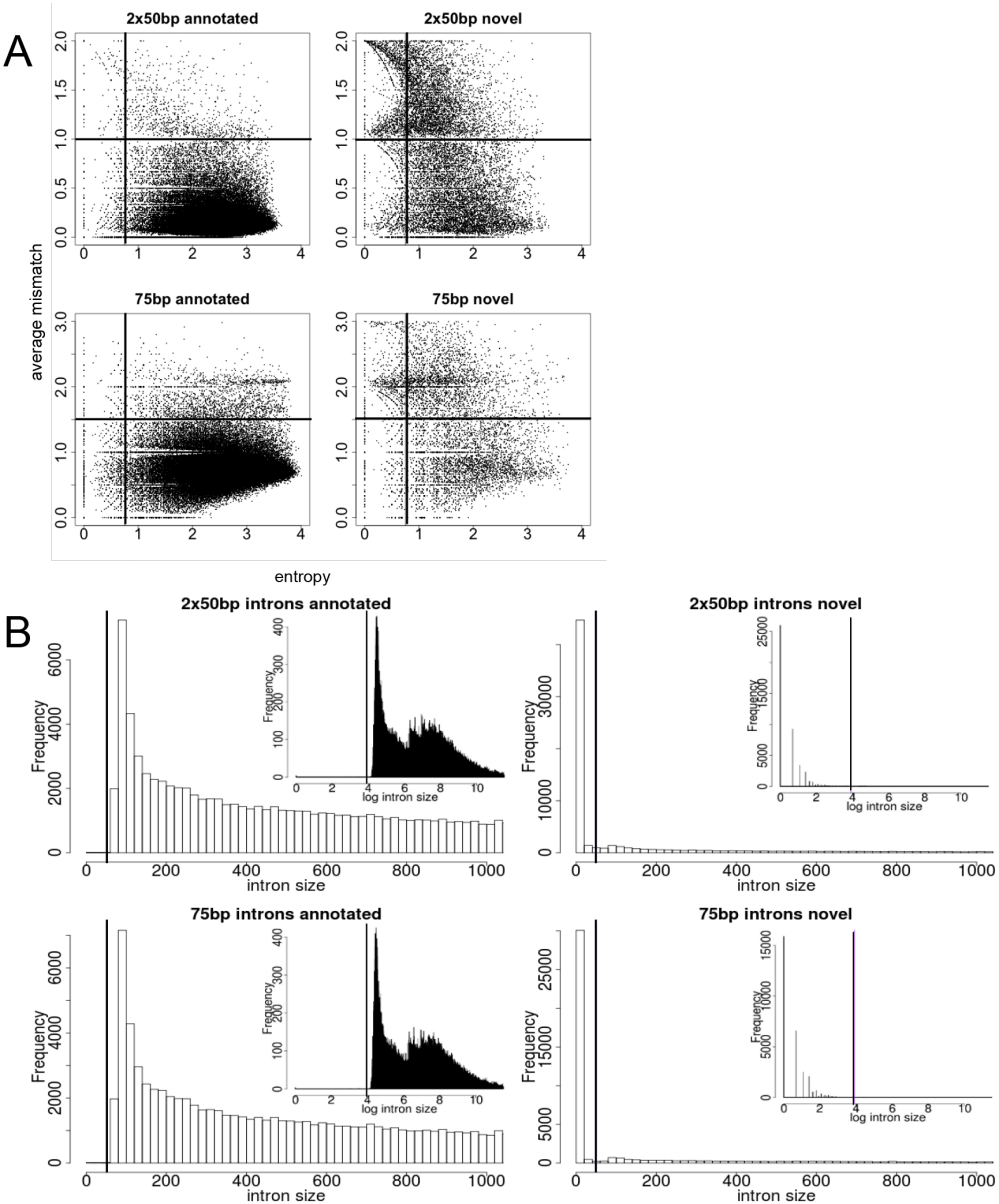
Plots for filtering splice junctions. (A) An example, from heart, plotting entropy versus average mismatches for annotated and novel splice junctions. Selected thresholds (0.75 entropy and 1.5 or 1 average mismatches, paired and single-end, respectively) are indicated by the dark lines and the lower right quadrants retained for further splice junction analyses. (B) Plots are for annotated only and novel splice junctions across all tissues for both paired-end and single end data. A vertical dark line indicates the applied threshold of 50 nucleotides. The main graphs are zoomed in to <1000bp intron sizes, while the sub-graphs show all natural log scaled intron sizes.

Though the MapSplice alignment tool [19] is one of the best for junction recall and precision [20], we chose to not use default settings for splice junction filtering, but determine best settings empirically. Initial alignments identified 925,775 and 850,194 putative splice junctions from single and paired-end reads respectively, of which approximately 29% represented previously annotated splice junctions. We presumed annotated junctions were likely true positives and used their attributes as a basis to set stringency filters to remove false positives. Specifically, we separated annotated from unannotated splice junctions and plotted entropy [19] versus average mismatches of all reads spanning a splice junction (Fig 1A). This lead to the selection of entropy thresholds of ≥ 0.75 for both library types and average mismatch thresholds ≤ 1.5 and 1 for single end and paired-end alignments respectively. Keeping splice junctions separated as annotated or novel, lengths of potentially spliced genomic sequence defined by each junction (i.e. potential introns) were plotted as histograms (Fig 1B). This suggested filtering putative splice junctions that were less than 50 nucleotides in length, which are more likely small deletions rather than true splice junctions. This size selection is consistent with minimum lengths of known introns [21].

**Fig 2:**
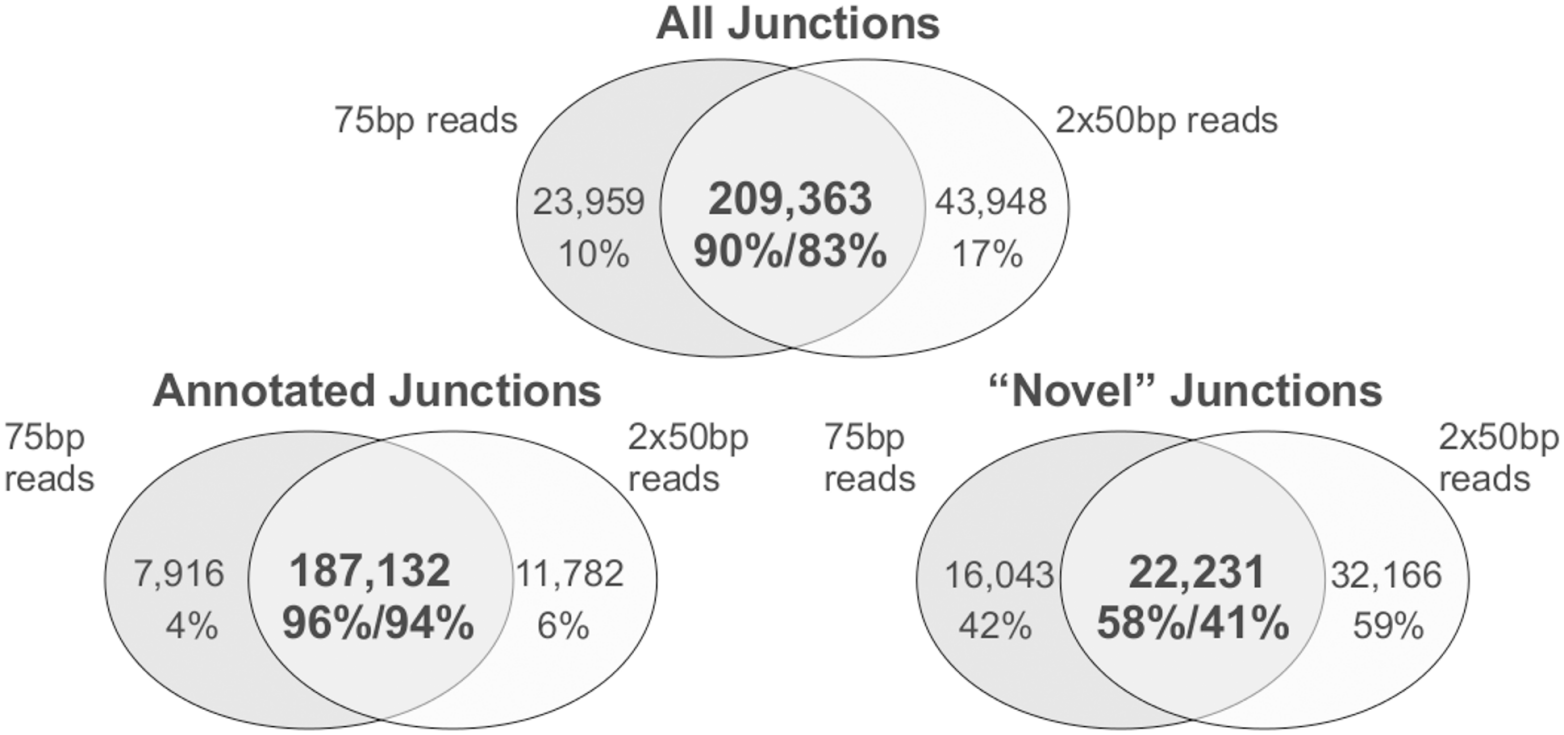
Concordance between the splice junctions identified by single end and paired-end RNA-seq reads. The three plots show all junctions (top), annotated junctions (bottom left), and novel junctions (bottom right).

Applying all of these filters removed 94.3% and 90.8% of the unannotated single and paired-end junctions, respectively, while retaining 75.4% and 76.9% of respective annotated single and paired-end junctions. Overall, this reduced the original number of putative splices 3-fold, while substantially increasing the percentage that had been previously annotated. These filtering methods resulted in 233,322 (84% annotated) single end and 253,311 (79% annotated) paired-end splice junctions. Approximately 87% of these splice junctions are concordant between paired-end and single end data (Fig 2). A substantial majority (95%) of the annotated junctions overlap between libraries, whereas 22,231 (50%, supplied as S1 File) overlap between the unannotated junctions. Of the unannotated splice junctions, 18–19% had both annotated donor and acceptor sites, but were just found in a new combination (Table 1). Additionally, approximately half of the unannotated splice junctions had either a splice donor or acceptor site annotated, but not the other (Table 1).

**Table 1:** Unannotated splice junction classifications.

	**Both Donor and Acceptor Annotated, but not as pair**	**Either Donor or Acceptor Annotated**	**Neither Donor or Acceptor Annotated**
Single end reads	6,827	18,222	13,225
Paired-end reads	10,134	23,852	20,411

Indicated are the number of unannotated splice junctions and if they have both, one, or no annotated splice donor/acceptor sites.

### Gene and splice junction distributions

**Fig 3:**
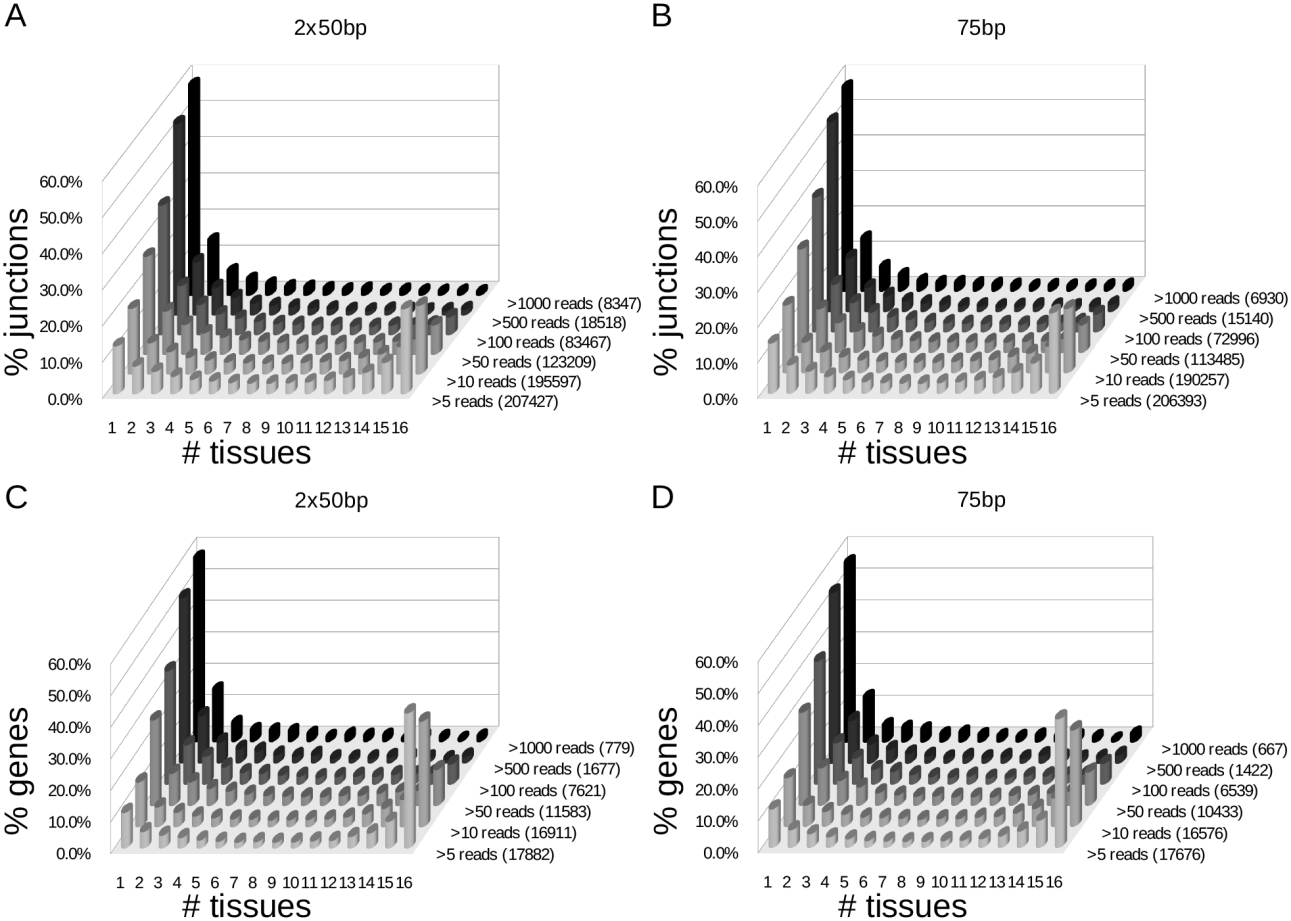
Gene and splice junction distribution. The percentage of genes or splice junctions found in the indicated number of tissues over a set threshold of reads from paired-end (2x50bp) and single end (75bp) reads. In parenthesis is the total number of genes or splice junctions found over a set threshold of reads.

Normalized expression levels for splice junctions and genes were determined. Genes and splice junctions were defined as expressed in a tissue when they were found over a set threshold of 5, 10, 50, 100, 500, or 1000 reads, equaling a range of RPKMs from approximately 1 (the minimal level for protein detection [22]) to 200. The tissue distribution of expression patterns shifted as the threshold value was changed (Fig 3). At higher levels of expression, most genes and junctions are tissue-specific. Lowering the threshold results in a progressive shift to a broader distribution pattern ranging from highly tissue-specific (expressed in only 1 tissue) to being present in all 16 tissues. Interestingly, the profiles for gene expression and splice junction expression do not change at a concurrent rate. Instead, the data demonstrate that splice junctions have a higher level of tissue-specificity. For example, at an expression threshold of >10 reads, 18% of the splice junctions were tissue-specific compared to only 14% of the genes. This relative difference was reversed at the other extreme, with 33% of genes, but only 19% of splice junctions expressed in all 16 tissues.

**Fig 4:**
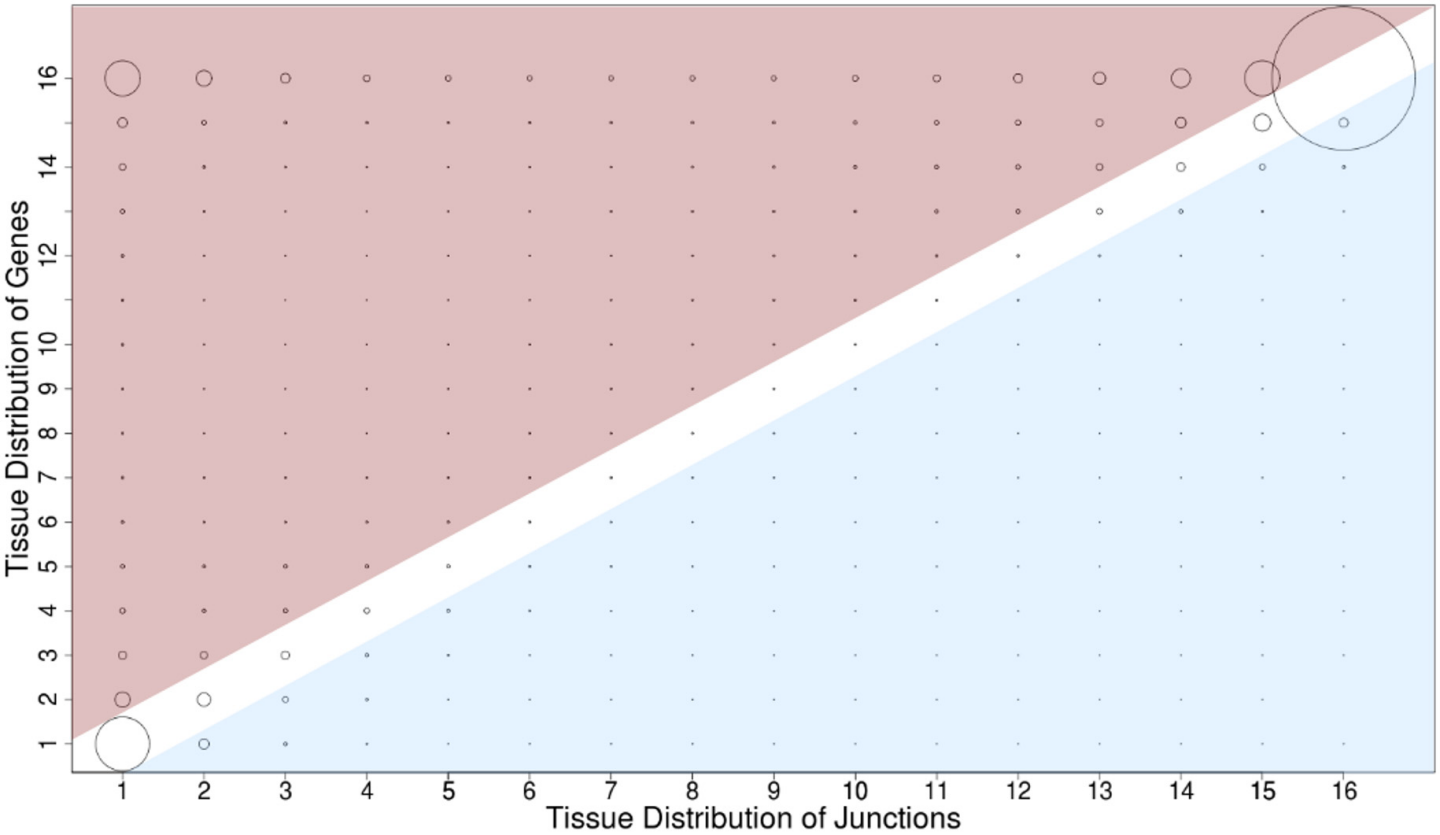
Gene and splice junction expression relations. Splice junctions from paired-end data have been assigned to genes and filtered for expression in tissues. Circle size depicts the number of junction-gene pairs. Plotted on the x-axis is the number of tissues a splice junction is found in and plotted on the y-axis is the number of tissues the corresponding gene is found in. Red regions indicated the gene is expressed in more tissues than the junction. Blue regions indicate the junction is found in more tissues than the gene, a rare event but possible due to threshold based analyses. Circles with a center in neither the blue or red region have their genes and junctions present in an equal number of tissues.

Tissue specificity relationships can be further analyzed by considering the expression pattern of individual splice junctions in the context of the expression pattern for their corresponding gene locus (Fig 4). Requiring a sequencing depth of >10 reads for both junctions and genes resulted in 191,730 junction-gene pairs in paired-end data. The largest two groups were junction-gene pairs expressed in all 16 tissues (32,997 pairs) or in only a single tissue (12,382 pairs). Surprisingly, however, the third largest group (representing 8,116 junction-gene pairs) contains splice junctions that were expressed in only one tissue from a gene expressed in all 16 tissues. The junction-gene discordance illustrates that a gene expressed broadly across many tissues can often contain highly tissue restricted exon splices. Indeed, at least one tissue-specific junction was found in approximately 67% of multi-exon genes expressed in all 16 tissues (Table 2). Overall, approximately 65% of total expressed multi-exon genes (9,942 of 16,102 single end genes and 11,264 of 16,411 paired-end genes) contain at least one tissue-specific splice junction.

**Table 2:** Genes with tissue restricted splice junctions.

	**# genes**	**# genes with a tissue restricted splice junction**
expressed	16,411	11,264 (69%)
expressed in >1 tissue	14,173	9,357 (66%)
expressed in 16 tissues	5,548	3,660 (66%)

Indicated are the number of multi-exon genes expressed and the number containing at least one tissue-specific splice junction from paired-end data.

### Tissue-specific genes and splice junctions

**Fig 5:**
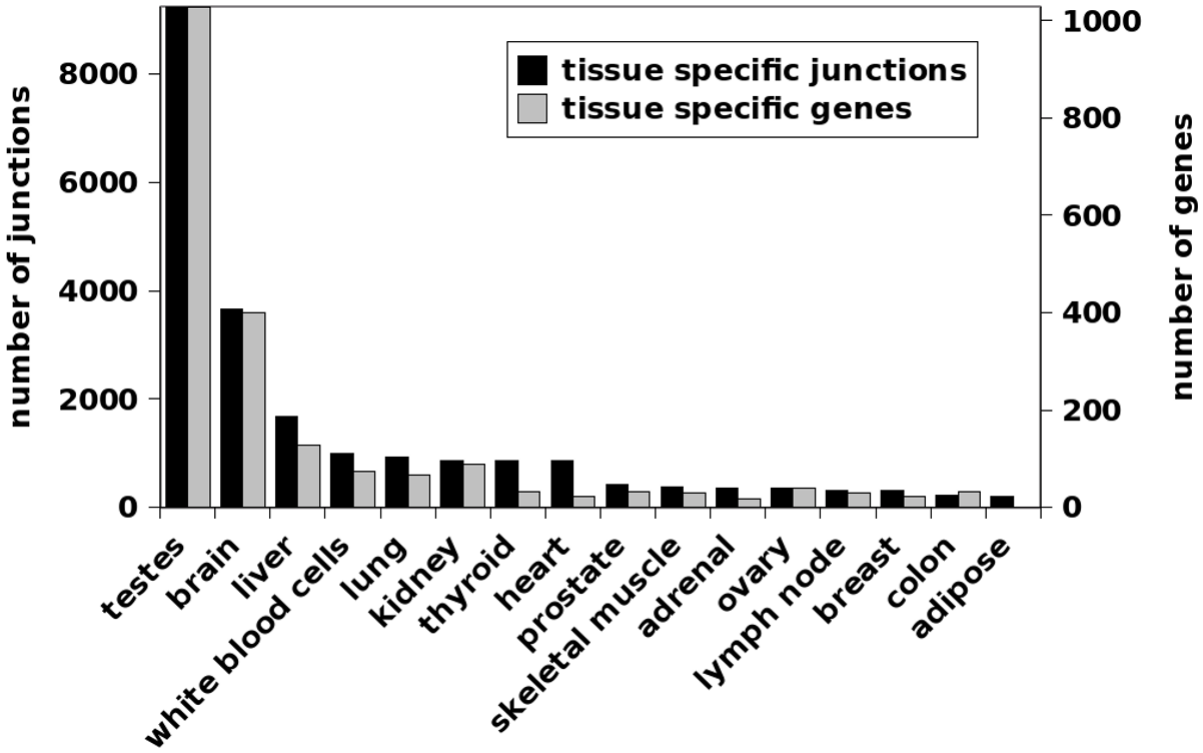
The number of splice junctions and genes specific to each tissue. Maximum values of the y-axis are set to the number of junctions/genes found in testes (9,234 splice junctions and 1,028 genes).

To examine tissue-specific genes and splice junctions, only genes and splice junctions found in both the single and paired-end data were used. Of these 21,650 tissue-specific splice junctions, the highest number was found in testes, followed by brain (Fig 5). Tissue-specific genes show a very similar distribution, with testes and brain having the highest number of tissue-specific genes (Fig 5). For visualization, the tissue-specific splice junctions have been placed in a bed format which can be uploaded as a custom track into the UCSC genome browser [23] (S2 File).

**Fig 6:**
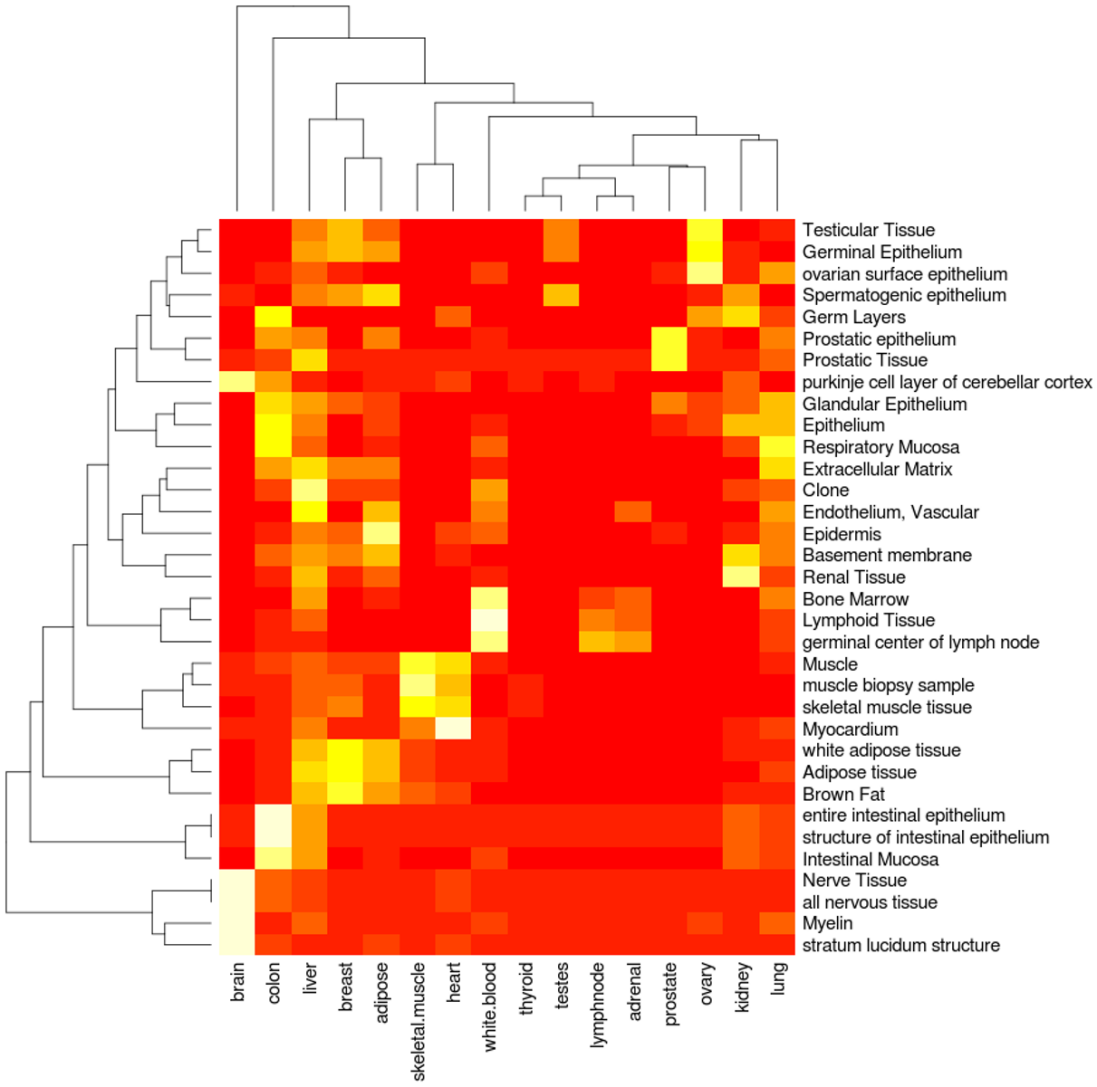
Text mining validation. Text mining results indicating the relationship between genes containing the tissue-specific RNA-seq splice junctions in each tissue (x-axis) and top tissue concepts (y-axis).

For validation of tissue specificity, text-mining demonstrated that genes containing tissue-specific splice junctions relate to appropriate tissue concepts (Fig 6) and GO [24, 25] biological processes (S1 Fig). We found high association between genes with tissue-specific splice junctions and relevant tissue concepts, such as “Muscle”, “muscle biopsy sample”, and “skeletal muscle tissue” for skeletal muscle. Relations to GO biological processes also showed many expected associations, such as “Myogenesis”, “muscle cell differentiation”, and “myoblast differentiation” for skeletal muscle. This illustrates literature support for the relation between the genes containing tissue-specific splice junctions and the appropriate tissues.

## Discussion

We have used Bodymap 2.0 RNA-seq data from 16 different human tissues to perform qualitative and quantitative analyses of tissue-restricted gene expression and splicing. Results were overall consistent with previous studies. We found more ubiquitously expressed genes (5,657 from single end and 5,016 from paired-end reads at a threshold of 10 reads) than found in a comparable study (3,510 at a similar threshold)[26], though Ramskold *et al*. evaluated a greater number of tissues (24) which potentially explains the increase. The number of tissue-specific genes in another study [27] also showed the highest number in testis (946, vs 1,028 in this manuscript) and brain (486, vs 401 in this manuscript). Similar to previous studies of differential exon use [28] and exon-skipping events [29], we observed the highest number of tissue restricted splice junctions in testes and brain (Fig 5). Overall, evaluating the number of tissues that genes or splice junctions (i.e. introns) are expressed in identifies a characteristic U-shaped pattern (Fig 3) [27, 29, 30]. Therefor most genes or splice-junctions are expressed either ubiquitously or are tissue-specific.

The threshold for expression is also informative. A high threshold of expression captures tissue biomarker genes. Relative differences become apparent, however, when the threshold is lowered. For example, at a threshold of 10 reads the largest gene category is those expressed in all 16 tissues, consistent with many shared biological processes in all cell types. In contrast, the splice junction distribution profile has almost equal levels of single and 16 tissue categories, suggesting expression of particular genomic loci is less informative than the qualitative structure in determining tissue specificity. At low levels of gene expression, it may be easier to detect the contiguous exon sequence of genes as compared to the exon junction sequences. To eliminate the possibility that this type of detection artifact was confounding our results and interpretation, the ratio of detectable splice junctions to detectable genes across all tissues from Fig 3 was calculated for each of the thresholds analyzed (S1 Table). If detection was indeed biased towards the detection of gene sequences over exon junction sequence at low expression levels, we would expect the calculated ratio to increase as the threshold of detection increased. What we find, however, is that the ratio remains stable across all thresholds, indicating that our results and interpretation are not influenced by this detection artifact.

Based on this, further evaluation of the relationship between tissue restricted splice junctions and tissue restricted gene expression was performed. If a gene is expressed in only a single tissue, then by extension the splice junctions in processed mRNAs from that gene will be in only a single tissue as well. This is reflected in the 1,1 circle of the matrix in Fig 4. Somewhat unexpectedly, a large number of tissue restricted splice junctions were found that are actually encoded by genes with the broadest (all 16 tissues) pattern of expression. Indeed, 8,116 junction-gene pairs were in this category. These data predict the extent to which widely expressed genes generate highly tissue restricted protein variants, presumably with tissue restricted functions. By extension, a major mechanism that achieves unique structural and functional features of individual cell types and tissues appears to be restricted patterns of primary transcript splicing from widely expressed gene loci. Thus, these highly restricted splice junctions have the potential to be sensitive molecular markers of tissue specificity to elucidate cell type, developmental stage, and/or pathology that would not have been identifiable through an analysis limited to only the total level of expression from each gene locus.

The Human Bodymap 2.0 dataset has shorter read lengths (1 x 75bp and 2 x 50bp) than the current standards (typically 2 x 100bp). However, this shorter read length likely has little affect on sensitivity of junction discovery and only a minor drop (∼3%) in specificity [19]. The dataset also does not contain biological replicates of the 16 tissues analyzed, which brings forth certain limitations. In the absence of biological replicates, there is no direct way to demonstrate that splice junctions categorized as tissue restricted are not in actuality specific to the donor of the individual tissue sample. This limitation will be addressed going forward as additional samples are analyzed by RNA sequencing, such as those being generated by the GTEx Consortium [27]. However, previous analyses have shown variation in gene expression and splicing to be low within populations [27, 31].

The separate analysis of the single-end and paired-end reads does, however, enable evaluation of the two datasets as technical replicates. The splice junctions identified by both libraries displayed high overlap (approximately 87%, Fig 2). They also show similar patterns of tissue specificity at the gene and splice junction levels (Fig 3), and expression of multi-exon genes with at least one tissue-specific junction (8,468 genes overlapping out of 9,942 single end and 11,264 paired-end genes). However, the level of concordance was much higher for annotated splice junctions (approximately 95%) than for the 22,231 novel splice junctions identified (approximately 50%) (Fig 2). A portion may reflect false positives, but the main reason for the difference is likely a failure to meet the entropy threshold in both datasets due to lower levels of expression. When we evaluated the number of annotated splice junctions above our thresholds in one library compared to all the junctions (i.e. no threshold) in the other library, we found 99.6% of 198,914 filtered splice junctions from pair-end reads in the 258,658 unfiltered single end data and 99.7% vice-verse (195,048 filtered splice junctions from single end reads in 258,812 unfiltered data from pair-end reads). For novel junctions, these values increased from 40.9% and 58.1% when comparing the overlap of filtered junctions only, to 72.8% and 83.8%, respectively, when looking at filtered-unfiltered overlaps. This indicates approximately 29% of novel junctions were detected, but were likely just under the threshold in the other library, as opposed to 5% of annotated junctions. Considering a genome as highly studied as human, it is not surprising that previously undiscovered splice junctions in normal tissues would tend to be expressed at lower (i.e. bordering threshold) levels.

In conclusion, we identified 209,363 human splice junctions, of which 10.6% were previously not annotated and 10.3% were expressed in a tissue restricted pattern. Tissue-specific alternative splicing was found to be widespread, occurring in approximately 65% of expressed multi-exon genes. Interestingly, many tissue restricted splice junctions are present not only in tissue restricted genes, but also in widely expressed genes.

## Materials and Methods

### RNA-seq read generation

We used the Human Bodymap 2.0 project RNA sequencing data which is distributed by Illumina and publicly available through the European Nucleotide Archive [32] (Accession #ERP000546). In summary, these RNA-seq samples were generated from 16 human adult tissues, including adipose, adrenal, brain, breast, colon, kidney, heart, liver, lung, lymph node, prostate, skeletal muscle, white blood cell, ovary, testes, and thyroid. Each tissue was from a different individual and presumed normal (i.e., not linked to the donor’s disease or cause of death). Standard Illumina mRNA-seq library preparations were performed to isolate poly-A selected mRNA. Each sample was then sequenced by Illumina on a single lane of a HiSeq 2000 for one run of 75bp single end reads and one run of 2 x 50bp paired-end reads (insert size approximately 210bp).

### Alignments

All 75bp single end reads and 2 x 50bp paired-end reads were aligned to the human reference genome (hg19 downloaded from UCSC [23]; chromosomes 1–22, X, and Y) using MapSplice v1.14.1 or v.1.15.2 [19]. The alignment was performed without annotation. The setting to identify non-canonical in addition to the traditional canonical splice sites was utilized. The minimum mapping length was set to the read length. In addition, the following non-default parameters were invoked: the ability to detect fusion events, mismatches permitted during remapping were set to a maximum of 2 nucleotides for paired-end reads and 3 for single end reads, and the maximum intron size was set to 100,000bp. Also, the option to run Bowtie [33] over 8 threads was used to decrease run times.

### Splice junction observations

The alignments were converted to putative splice junctions using the MapSplice “newSAM2junc” module. However, as opposed to applying default cutoffs for junction discovery with the MapSplice “filterjuncbyROCarguNonCanonical” module, we determined filters empirically. Junctions were first annotated against known intron annotation from UCSC [23, 34–36] and Ensembl [37] (downloaded from the March 2011 UCSC website tables). Plots were then made from the calculated average entropy against the average number of nucleotide mismatches in the full set of aligned reads at each putative splice junction (Fig 1A). Annotated junctions were considered the gold standard. Therefore, using these plots, thresholds for entropy and mismatches were set to maintain annotated junctions, while at the same time excluding poorly supported unannotated junctions, and hence limiting the number of false positives. Histograms of splice junction (i.e. intron) sizes were plotted to determine an additional threshold value, which was then used to distinguish small deletions from true splice junctions (Fig 1B).

To report previously unannotated splice junctions and make them easily viewable in the UCSC Genome Browser, novel splice junctions have been converted into a bed format file (S1 File). This file has been designed to show the last bp of the upstream exon and first bp of the downstream exon as a thicker box, connected by a thin line.

### Gene and splice junction expression patterns

Raw splice junction expression values were determined by the number of reads aligning across a splice junction. Raw gene expression levels were determined by the average read coverage across Ensembl genes. This was done by converting SAM output files from every tissue to pileup format with SAMtools [38], and then to bedGraph format with a custom script. These files were evaluated with Ensembl 61 protein-coding genes (retrieved through Biomart [37, 39]) and the number of aligned reads across all exonic base-pairs totaled and divided by the gene length. Both raw junction and gene expression values were normalized to account for different numbers of aligned reads in each tissue. The target tissue’s raw values were multiplied times the average number of total reads across all tissues and divided by the target tissue’s total number of reads. This method provides a comparable value for both junction and gene expression values. This is similar to calculating RPKM [13], but normalizes on nucleotide coverage instead of read number, and the mean number of reads mapped across samples instead of a million mapped reads, resulting in approximately 6.4 and 5.1 times higher for paired-end and single end data, respectively. The number of splice junctions and genes expressed in a given number of tissues were then analyzed at different set thresholds (5, 10, 50, 100, 500, or 1000 reads). Splice junctions and genes were categorized as tissue-specific when expressed above the threshold in only one of the sixteen tissues.

Remaining analysis was performed using a threshold of 10 reads. Using this threshold, splice junctions were paired to a gene if they matched an Ensembl annotated splice donor and/or acceptor site. Due to overlapping genes or an acceptor site in one gene with a donor site in another gene, some splice junctions could be assigned to multiple genes. These multi-gene junctions were excluded from the analysis. In addition, lists were generated for each tissue of tissue-specific splice junctions and genes that were found in both the paired-end and single end data. All 16 tissue-specific splice junction lists were taken together and converted to a single bed format file (S2 File) as described above, including using the specific tissue in the name field.

### Text mining for tissue validation

To investigate whether tissue-specific splice junctions were from genes with known tissue associations, text mining was performed with the tool Anni 2.1 [40]. Anni can take two sets of concepts, such as a user supplied list of genes and either GO biological processes or tissue (both supplied as predefined concept sets in Anni), and can derive a matrix with association strengths for the concepts in these sets based on the matching of text-mining derived concept profiles. For our gene list, we took all tissue-specific splice junctions (due to computational constraints, brain, liver, and testes were randomly reduced to 1000 junctions) and converted them to HGNC IDs (reporting unique results only) with Ensembl 61 Biomart. These were loaded as a new concept set into Anni and matched against the concept sets for GO biological processes and tissues. To identify processes common to all tissues, 1000 random protein coding genes were selected and run through as an additional concept set.

The top 5 concepts for GO biological processes and tissue annotation were selected for each RNA-seq tissue sample and their summarized matching scores extracted for all RNA-seq samples. Since these sum scores are partially based on the number of concepts in a set and some tissue-specific sets had more concepts than others, the scores were normalized based on the number of concepts in our concept sets (i.e. the sum score per GO/tissue concept was divided by the number of RNA-seq tissue concepts). The equivalent normalized random genes’ sum score was subtracted from the RNA-seq tissue sample’s sum score per GO/tissue concept (negative values were set to 0). These normalized scores were then plotted as heat maps using R statistical software.

## Supporting Information

S1 File Bed track of novel human splice junctions. These 22,231 previously unannotated splice junctions are found in both paired-end and single end data. This file is viewable in the UCSC Genome Browser.
(BED)

S2 File Bed track of human tissue restricted splice junctions. This file is viewable in the UCSC Genome Browser.
(BED)

S1 Fig Heat map of GO biological processes in relation to genes containing tissue restricted splice junctions. Plotted are relationship values from text-mining for top 5 GO biological processes for each tissue after normalization and background filtering.
(PNG)

S1 Table Ratio of detectable junctions to detectable genes. (PDF)
