# Supplementary figures and images for: Tissue Restricted Splice Junctions Originate Not Only from Tissue-Specific Gene Loci, but Gene Loci with a Broad Pattern of Expression
Source: PLoS One. 2015 Dec 29;10(12):e0144302. doi: 10.1371/journal.pone.0144302 (PMC4695084; doi:10.1371/journal.pone.0144302)

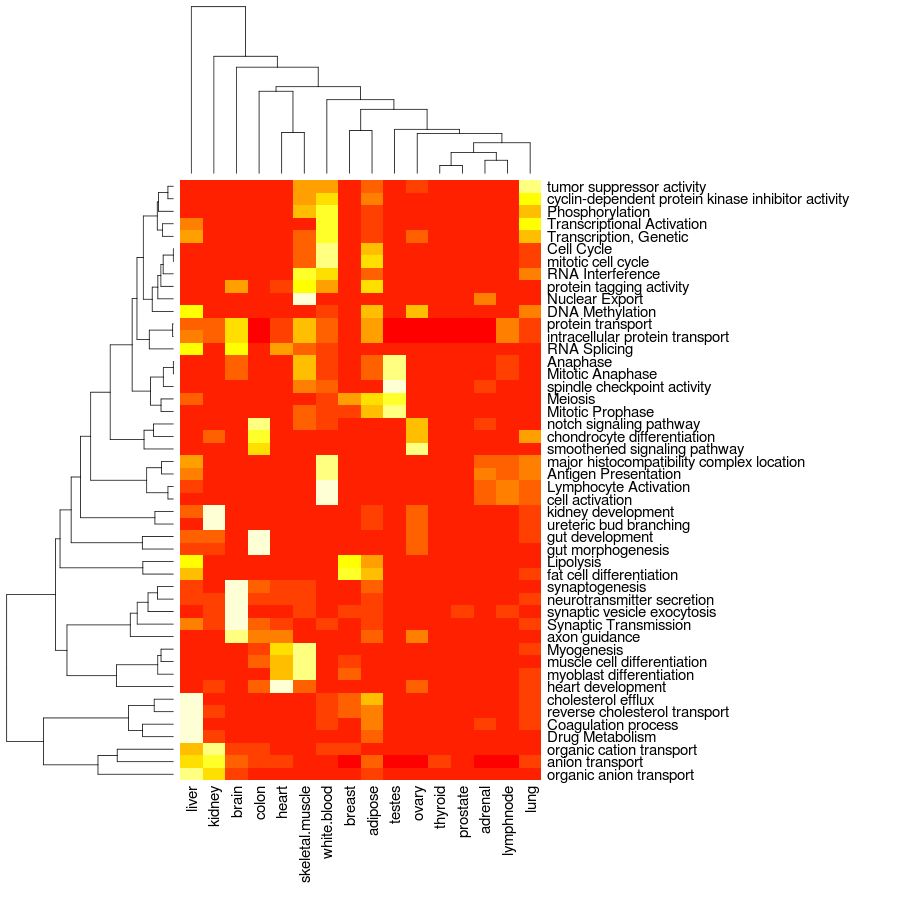

Supplement: S1 Fig — Plotted are relationship values from text-mining for top 5 GO biological processes for each tissue after normalization and background filtering. (PNG) [file pone.0144302.s003.png]
