# Supplementary material for: Tissue Restricted Splice Junctions Originate Not Only from Tissue-Specific Gene Loci, but Gene Loci with a Broad Pattern of Expression
Source: PLoS One. 2015 Dec 29;10(12):e0144302. doi: 10.1371/journal.pone.0144302 (PMC4695084; doi:10.1371/journal.pone.0144302)
Supplement: S1 Table — (PDF) [file pone.0144302.s004.pdf]

Ratio of Junctions to Genes

Read Threshold	>5 reads	>10 reads	>50 reads	>100 reads	>500 reads	>1000 reads
Single end	11.68	11.48	10.88	11.16	10.65	10.39
Paired-end	11.60	11.57	10.64	10.95	11.04	10.72
